# OP23 Developing A Personalized Decision Aid Incorporating A Discrete Choice Experiment: A Case Study In Ulcerative Colitis

**DOI:** 10.1017/S0266462325100810

**Published:** 2025-12-29

**Authors:** Nyantara Wickramasekera

## Abstract

**Introduction:**

Choosing the optimal ulcerative colitis treatment is complex, given the range of medical and surgical options with varying side effects and effectiveness. Decision aids can improve patient choices, but current tools lack personalization. To address this, we developed a personalized decision tool using a discrete choice experiment (DCE) to help patients make informed decisions about medical or surgical treatments.

**Methods:**

An online DCE survey was developed containing competing treatment profiles described using all important aspects of the treatment (effectiveness, side effects, family planning). Patients (n=300) with ulcerative colitis were asked to consider the benefits and disadvantages of each treatment profile and select the treatment that they would choose. The DCE data were analyzed using mixed logit and latent class models. The model results were integrated into an online decision aid using a Shiny application.

**Results:**

R Shiny was successfully used to enable the real-time personalization of DCE results. The developed decision aid contained two aspects of personalization. First, attribute importance scores showed the treatment characteristics that mattered most to patients based on their DCE choices. Second, a “best-match” treatment that aligned with their preferences was provided from uptake rate calculations. User testing of the developed decision aid is ongoing. However, initial feedback from patients has been positive.

**Conclusions:**

A key challenge in developing personalized decision aids is providing real-time, tailored recommendations based on individual preferences. This study demonstrated the feasibility of integrating DCE methods into personalized decision aids for ulcerative colitis. By tailoring treatment recommendations to individual patient preferences, this tool has the potential to empower patients, reduce decisional conflict, and enhance shared decision-making between patients and clinicians.

